# Cerebral Hyperperfusion Syndrome Presenting As Status Epilepticus Following Carotid Endarterectomy

**DOI:** 10.7759/cureus.20551

**Published:** 2021-12-20

**Authors:** Alicia M Edwards, Caleb R Birchler, Sean Park, Jennifer M Baker, Robert G Molnar

**Affiliations:** 1 Vascular Surgery, McLaren Flint Hospital, Flint, USA; 2 Surgery, Michigan State University, East Lansing, USA; 3 Vascular Surgery, Michigan Vascular Center, Flint, USA

**Keywords:** cerebral edema, status epilepticus, carotid endarterectomy, seizure, hyper-perfusion

## Abstract

Cerebral Hyperperfusion Syndrome (CHS) is a rare syndrome, commonly described as a prodrome of symptoms including a severe ipsilateral headache, focal neurological deficits, intracerebral hemorrhage, and occasionally includes seizures or encephalopathy. Our case involves a 76-year-old man who underwent a left carotid endarterectomy (CEA) for symptomatic high-grade stenosis of his left carotid artery. Post-operative day one, the patient was seen and examined in the early morning and found to be doing well, with blood pressures well-controlled and at his neurologic baseline. Three hours later, he was reported to have a sudden spike in his blood pressure and was experiencing focal motor seizures involving the right arm and face, both of which were unrelieved by anti-hypertensives and anti-seizure medications. The patient subsequently developed worsening respiratory function requiring intubation for status epilepticus. Repeat head and neck imaging with CT, CT angiography, and MRI demonstrated the known previous subacute infarct with new cerebral edema, patent carotid arteries bilaterally, and no acute infarct or intracerebral hemorrhage.

While CHS is a rare syndrome with well-documented symptomatology, we present a unique case in which focal motor status epilepticus was the only presenting symptom in a patient who otherwise meets the criteria of CHS based on radiographic evidence of cerebral edema following an elective CEA.

## Introduction

Cerebral Hyperperfusion Syndrome (CHS), or cerebral reperfusion injury, is a rare syndrome that has been described as a clinical constellation of symptoms of severe headache, ipsilateral to the side of reperfusion, focal neurological deficits, possible intracerebral hemorrhage, and in rare cases, with seizures or encephalopathy [1]. The incidence of CHS or CHS with intracerebral hemorrhage (ICH) following carotid endarterectomy (CEA) has been reported as 1.9% and 0.37%, respectively [2]. In a 2011 meta-analysis, Bouri and colleagues proposed four criteria to standardize the classification of post-carotid endarterectomy CHS: (1) occurring within 30 days of CEA, (2) radiographic evidence of hypoperfusion, (3) clinical features including a new headache, seizure, hemiparesis, GCS <15, or radiographic evidence of edema or ICH, and (4) no evidence of a new acute cerebral ischemia, carotid occlusion, or an identified metabolic or physiological cause [3]. The edema associated with CHS is generally considered to be reversible, but with a worsening prognosis when CHS is associated with intracerebral hemorrhage, in which approximately 30% of the patients remain partially disabled with an additional 50% resulting in mortality [2]. The major risk factors associated with development of CHS or ICH post CEA are postoperative hypertension (systolic blood pressure >180 mmHg), history of coronary artery disease, contralateral stenosis of >70%, and an ipsilateral stroke within the past month [1,4]. It has likewise been suggested in previous studies that prevention of CHS is best obtained through proper control of blood pressure, timing of the CEA in relation to a TIA or stroke, and careful selection of anesthetic to be used, with consideration of the anesthetic's impact on intracerebral vasculature [1,2].

## Case presentation

A 76-year-old male with a past medical history of hypertension, chronic obstructive pulmonary disease (COPD), and left hemispheric ischemic stroke/cerebral vascular attack (CVA) with residual aphasia and right-hand numbness underwent a left CEA approximately four months after the onset of his index CVA. Preoperatively, the patient was found to have a right carotid artery with less than 10% stenosis and a left carotid artery with 99% stenosis in the area between C2 and C3 levels. The CEA was uneventful without intraoperative or immediate postoperative complications, and his systolic blood pressure was well controlled and always less than 160mmHg. 

On postoperative day one, the patient was initially evaluated in the morning and found to be neurologically intact, at his baseline, with his systolic blood pressure well-controlled below 160mmHg. Approximately three hours later, he was noted to have a sudden rise in his systolic blood pressure, to 200mmHg, which was unresponsive to multiple intravenous pushes of his nicardipine drip, as well as a new onset of uncontrollable focal motor seizures involving his right arm and face. At this time, the patient was able to follow commands and had no other lateralizing signs or focal neurological deficits noted during a stroke evaluation (National Institutes of Health Stroke Scale (NIHSS) 0). Despite multiple doses of midazolam and lorazepam at this time, the patient continued to have focal motor seizures of his right arm and face along with a subsequent decline in his respiratory status; thus, the decision was made to intubate him for airway protection in the setting of refractory focal status epilepticus. 

**Figure 1:**
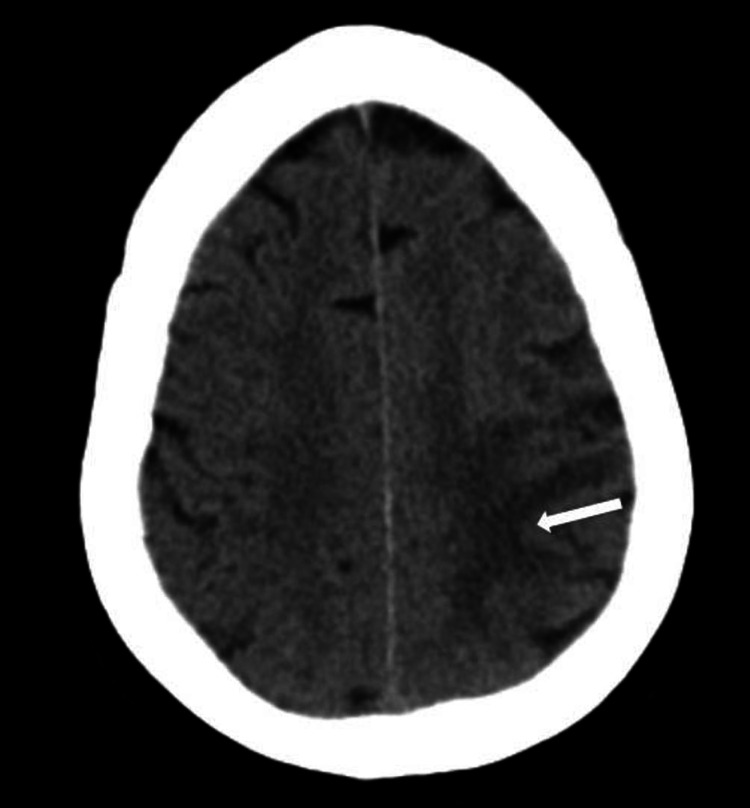
CT Head without contrast, demonstrating subacute infarct and cerebral edema (arrow) CT: computed tomography

**Figure 2:**
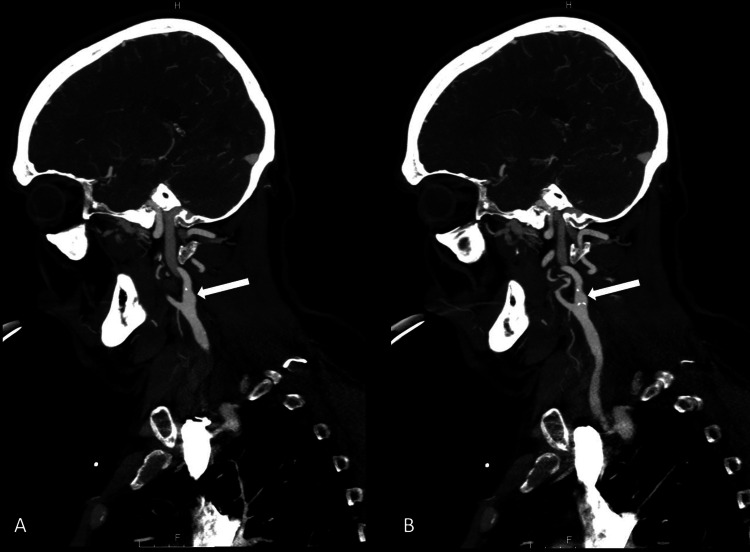
CTA Head and Neck: two sequential images A & B, using 5mm slices, demonstrates widely patent left ICA (arrow), post CEA CTA: computed tomography angiography; ICA: internal carotid artery; CEA: carotid endarterectomy

**Figure 3:**
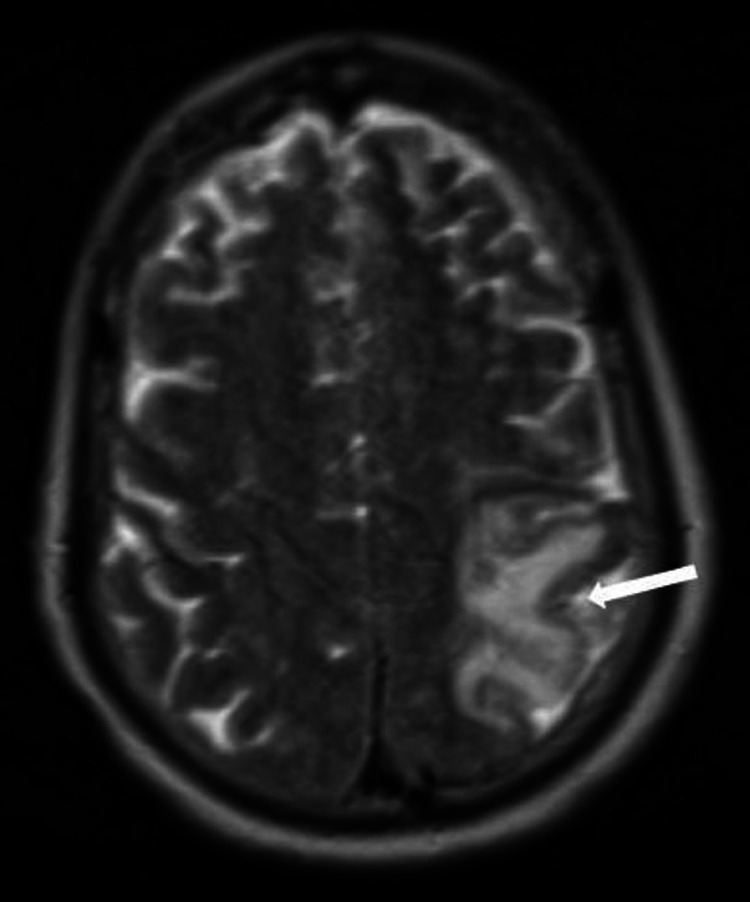
MRI brain with subacute infarct and associated edema (arrow) around left MCA. No evidence of hemorrhage or new acute infarct is demonstrated MRI: magnetic resonance imaging; MCA: middle cerebral artery

Multiple imaging studies were obtained from the initial symptom onset and subsequently throughout the hospital stay. Initial CT head was unremarkable for a new acute CVA but did demonstrate left hemisphere edema (Figure 1). No major occlusion or hemorrhage was found on initial or subsequent CT angiography, and bilateral carotid arteries were noted to be widely patent (Figure 2). A carotid duplex exam, which was performed after the patient was intubated and sedated, also revealed widely patent left internal, external, and common carotid arteries. Subsequent MRI revealed mild edema in the left middle cerebral artery (MCA) region without evidence of any hemorrhage or acute infarct (Figure 3). Thus, the immediate and subsequent imaging in conjunction with the timing of the clinical presentation confirmed the suspected diagnosis of CHS.

For the remaining hospital stay (15 days), the patient was monitored with continuous EEG while remaining intubated and sedated with propofol and high doses of anti-seizure medications, including Vimpat®, Dilantin®, Keppra®, and a midazolam drip. Multiple attempts were made to reduce the midazolam drip rate with recurrent seizures occurring at rates below 20cc/hr. On postoperative day 14, the patient’s family decided to transition to comfort care measures, and the patient subsequently passed on postoperative day 15, and no autopsy was performed.

## Discussion

This case is unique in that refractory focal status epilepticus was the only clinical indication towards a diagnosis of CHS in a patient with no other risk factors to suspect CHS. The patient did not have contralateral carotid stenosis, his previously known ipsilateral stroke had occurred more than one month prior to surgery, he did not have a history of coronary artery disease, and his blood pressure was well controlled postoperatively up until the focal motor seizure activity began, thus minimizing potential risks of developing CHS. 

He underwent a left CEA for a left hemispheric ischemic stroke with residual aphasia and right-hand numbness which had occurred four months prior to surgery. One could speculate that his brain tissue had since adapted and developed collateral blood flows to the ischemic area, and the increased blood flow from the CEA resulted in cerebral edema. However, repetitive head/brain CT imaging throughout the hospital course demonstrated cerebral edema in the setting of a subacute stroke and did not demonstrate any intracerebral hemorrhage nor any areas of acute cerebral ischemia, which are two imaging findings classically associated with reperfusion injury. Additionally, the patient’s blood pressure was well controlled with anti-hypertensive medications, including their daily lisinopril and an as-needed nicardipine drip until approximately 10 minutes before the focal seizures began, at which time the systolic blood pressure rapidly rose to greater than 200 mmHg, and was unresponsive to any additional increase in the nicardipine drip-rate. With the patient’s prior history of CVA, the selection of anti-hypertensives could have been optimized. Specifically, using antihypertensive medications with vasodilatory effects (i.e calcium channel blockers, sodium nitroprusside, or angiotensin II inhibitors) is thought to increase the risk of CHS in patients with prior brain ischemia due to increased cerebral blood flow and thus increased perfusion [2]. Both labetalol and clonidine have been shown to be effective in treating blood pressure in patients with CHS, as neither increases cerebral blood flow. Labetalol is a mixed alpha- and beta-adrenergic antagonist that reduces the risk of reflex hypertension seen with the use of other pure beta-antagonists. However, our patient’s blood pressure was well-controlled until just prior to his focal seizures began, which could also indicate a different pathological process was contributing to the patient’s clinical presentation.

With regards to the timing of this patient’s CEA, the ideal timing of the revascularization procedure for secondary stroke prevention is based upon the severity of the initial stroke. According to the 2019 update of the American Heart Association and American Stroke Association “Get with The Guidelines” recommendations, carotid revascularization for minor, non-disabling strokes is reasonable to be performed within 48 hours to seven days of the index stroke [5]. However, larger strokes that resulted in residual and/or disabling symptoms, such as the one our patient originally experienced, are not recommended to undergo early revascularization procedures. These larger strokes are often still evolving during the subacute time period and thus have a higher risk of CHS or cerebral hemorrhage if revascularization is performed too early [2]. It is not uncommon for revascularization of these larger strokes to be performed four to six weeks or later after the index CVA, as was the situation for our case where the revascularization procedure occurred four months after the index CVA.

Finally, choice of anesthetic and surgical approach have been previously commented upon in the literature about their ability to contribute to the subsequent development of CHS. Specifically, the use of high dose volatile halogenated hydrocarbon anesthetics are thought to lead to CHS, whereas isoflurane, nitrous oxide, and propofol are all considered safe anesthetic options [2]. At our institution, the standard anesthetic agent is propofol subsequent to rapid sequence intubation, which has been noted to normalize cerebral blood flow and is a safe option for carotid revascularization procedures [2,6-7].

## Conclusions

This is the first case, to our knowledge, in the published literature in which a post-CEA patient presents with CHS purely in the form of focal status epilepticus. Cerebral hyperperfusion has been previously correlated to risk factors such as contralateral carotid stenosis, a recent ipsilateral stroke, coronary artery disease, and/or uncontrolled blood pressure; however, none of these risk factors were noted in our patient. Our patient did have an acute spike in his blood pressure, but only at the time that his focal seizures presented. We thus present this case to highlight that one should be alert to the sudden onset of focal motor seizures and subsequent refractory focal status epilepticus as a symptom of CHS after CEA; one which can occur in isolation. Despite our best efforts at prevention, this case highlights that instances of CHS may still occur without warning and may present with isolated focal motor seizures devoid of the more commonly recognized symptoms of CHS.
